# A Population Pharmacokinetic Study to Compare a Novel Empagliflozin L-Proline Formulation with Its Conventional Formulation in Healthy Subjects

**DOI:** 10.3390/ph17040522

**Published:** 2024-04-18

**Authors:** Xu Jiang, Kyung-Sang Yu, Dong Hyuk Nam, Jaeseong Oh

**Affiliations:** 1 Department of Pharmacology, College of Medicine, Yonsei University, Seoul 03722, Republic of Korea; kangwook@yonsei.ac.kr; 2 Department of Clinical Pharmacology and Therapeutics, College of Medicine, Seoul National University and Hospital, Seoul 03080, Republic of Korea; ksyu@snu.ac.kr; 3 Department of Biomedical Sciences, College of Medicine, Seoul National University, Seoul 03080, Republic of Korea; 4 Department of Chemical Research Laboratory, Chong Kun Dang Research Institute, Chong Kun Dang Pharmaceutical Corporation, Yongin 16995, Republic of Korea; namdh@ckdpharm.com; 5 Department of Pharmacology, College of Medicine, Jeju National University, Jeju 63243, Republic of Korea; 6 Clinical Research Institute, Jeju National University Hospital, Jeju 63243, Republic of Korea

**Keywords:** empagliflozin, empagliflozin L-proline, cocrystal, population pharmacokinetic modeling

## Abstract

Empagliflozin is a sodium–glucose cotransporter 2 (SGLT2) inhibitor that is commonly used for the treatment of type 2 diabetes mellitus (T2DM). CKD-370 was newly developed as a cocrystal formulation of empagliflozin with co-former L-proline, which has been confirmed to be bioequivalent in South Korea. This study aimed to quantify the differences in the absorption phase and pharmacokinetic (PK) parameters of two empagliflozin formulations in healthy subjects by using population PK analysis. The plasma concentration data of empagliflozin were obtained from two randomized, open-label, crossover, phase 1 clinical studies in healthy Korean subjects after a single-dose administration. A population PK model was constructed by using a nonlinear mixed-effects (NLME) approach (Monolix Suite 2021R1). Interindividual variability (IIV) and interoccasion variability (IOV) were investigated. The final model was evaluated by goodness-of-fit (GOF) diagnostic plots, visual predictive checks (VPCs), prediction errors, and bootstrapping. The PK of empagliflozin was adequately described with a two-compartment combined transit compartment model with first-order absorption and elimination. Log-transformed body weight significantly influenced systemic clearance (CL) and the volume of distribution in the peripheral compartment (V2) of empagliflozin. GOF plots, VPCs, prediction errors, and the bootstrapping of the final model suggested that the proposed model was adequate and robust, with good precision at different dose strengths. The cocrystal form did not affect the absorption phase of the drug, and the PK parameters were not affected by the different treatments.

## 1. Introduction

Type 2 diabetes mellitus (T2DM) is a chronic metabolic disease that is becoming more common globally. The global incidence of diabetes reached pandemic proportions in 2019 and increased from 9.3% in 2019 to 10.5% in 2021, and in South Korea, the prevalence of diabetes increased from 10.3% in 2021 to 13.9% in 2020 [1]. It is estimated that in 2040, there will be more than 640 million people with diabetes worldwide [2]. It is also becoming more common in young individuals [3].

Many antidiabetic drugs, such as sulfonylureas, biguanides, α-glucosidase inhibitors, dipeptidyl peptidase 4 inhibitors, and sodium–glucose cotransporter 2 (SGLT2) inhibitors, have been developed [4]. The research and development of optimized antidiabetic drugs are ongoing, and new small-molecule GLP-1 receptor agonists have been developed and are currently in clinical trials [5].

SGLT2 inhibitors, which were first developed in 2013, are a class of hypoglycemic agents that work by inhibiting glucose reabsorption in the kidneys, resulting in increased glucose excretion in the urine and decreased blood glucose levels [6]. Empagliflozin (Jardiance^®^), a representative SGLT2 inhibitor, was first launched in 2014 under license from Boehringer Ingelheim International GmbH, Ingelheim, Germany [7]. Empagliflozin is a commonly used SGLT2 inhibitor that has been shown to be effective at reducing blood glucose levels and improving cardiovascular outcomes in people with T2DM [8]. Recently, some new findings showed that empagliflozin can reduce brain pathology in Alzheimer’s disease and T2DM, and it can also mitigate cardiac hypertrophy and improve autoimmune myocarditis [9,10,11,12]. Pharmacokinetic (PK) studies of empagliflozin in healthy subjects have been conducted in many countries [13,14,15,16].

Currently, the cocrystallization of drugs with appropriate co-formers is becoming a promising approach for improving the [17] oral absorption, chemical properties, stability, solubility, permeability, and dissolution rate of drugs, to facilitate the development of pharmaceutical formulations [7,18,19,20,21,22]. One such example is Suglat^®^ (Ipragliflozin L-proline), a SGLT2 inhibitor which was developed as a cocrystal formulation with the co-former L-proline [23]. Furthermore, recently, a novel dapagliflozin di-L-proline cocrystal-loaded tablet was developed which aimed to solve the problems caused by the severe hygroscopic properties of dapagliflozin [24]. Empagliflozin L-proline, a newly developed cocrystallized formulation of empagliflozin with a co-former of L-proline, also known as CKD-370, was developed by Chong Kun Dang Pharmaceutical Corp. (Seoul, Republic of Korea). According to the preclinical studies, it was found that empagliflozin L-proline can be broken down to empagliflozin in the digestive system and be absorbed in the form of empagliflozin (data on file). Two comparative PK studies, one on CKD-370 and empagliflozin and another on CKD-370/metformin and empagliflozin/metformin fixed-dose combinations, were both conducted in 2019 [25,26]. The PK, safety, and tolerability of these two empagliflozin formulations were investigated through clinical studies.

Population PK analysis is known to be a useful tool for conducting more mechanistic assessments of PK properties and for more detailed explorations and evaluations of covariates. This approach can explain the variability between subjects and occasions and establish relationships between drug exposure and relevant PK parameters. This approach can facilitate the selection of dosing schedules and the development of strategies for dose individualization [27]. However, there has been no population PK study of empagliflozin L-proline to elucidate its PK characteristics compared to those of empagliflozin.

Based on these findings, this study aimed to develop a population PK model to compare the PK characteristics of empagliflozin L-proline with those of conventional empagliflozin formulations.

## 2. Results

### 2.1. Demographics and Datasets

A total of 27 subjects were included in each study. There were 22 males and 5 females in the first study, and 20 males and 7 females in the second study. A total of 864 plasma concentrations (432 plasma concentrations for each formulation) and a total of 968 plasma concentrations (485 plasma concentrations for empagliflozin and 483 plasma concentrations for empagliflozin L-proline) were obtained from the two studies, respectively, and used in the model’s development and evaluation. The characteristics of all included subjects are summarized in Table 1.

### 2.2. Base Model Development

**Figure 1:**
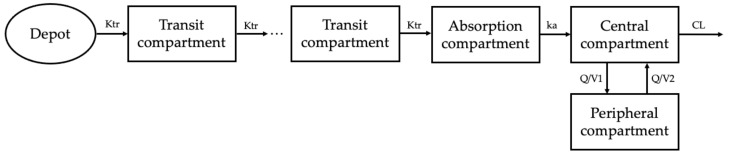
Empagliflozin population PK model. A two-compartment combined transit compartment model with first-order absorption and elimination best fits the observed empagliflozin plasma concentrations. Ktr, transit rate constant; ka, absorption rate constant; Q, intercompartmental clearance; CL, clearance.

A two-compartment combined transit compartment model with first-order absorption and elimination best described the data. The two-compartment model with lag time and the two-compartment model with transit compartment showed similar fits according to the GOF plots. By comparing the OFV and BIC values, a transit compartment model was chosen to explain the absorption process. The structural representation of the base model is shown in Figure 1. A proportional error model was found to best describe the residuals.

### 2.3. Covariate Analysis

Among the tested covariates (Table 1), only body weight was identified as a statistically and clinically significant covariate and was included in the final model. The covariate screening results showed that the inclusion of log-transformed body weight on clearance (CL) and the volume of distribution in the peripheral compartment (V2), sex on V2, and ALP on intercompartmental clearance (Q) significantly improved the model fit. ALP was excluded because it was not clinically relevant to Q, and sex on V2 was also excluded from the final model due to poor estimation precision (relative standard error greater than 70%). Finally, the effects of the IIV on the absorption rate constant (Ka), CL, V2, and IOV on the transit rate constant (Ktr), mean transit time (Mtt), Ka, and CL best described the data. The outputs of the final model are summarized in Table 2. The number of transit compartments was confirmed to be 4.79 using Formula (1). Most of the PK parameters for the final model were estimated with good precision (i.e., small % RSE), suggesting adequate reliability.

(1) $$\mathrm{Mtt}=\frac{n+1}{K_{\mathrm{tr}}}$$

### 2.4. Model Evaluation

**Figure 2:**
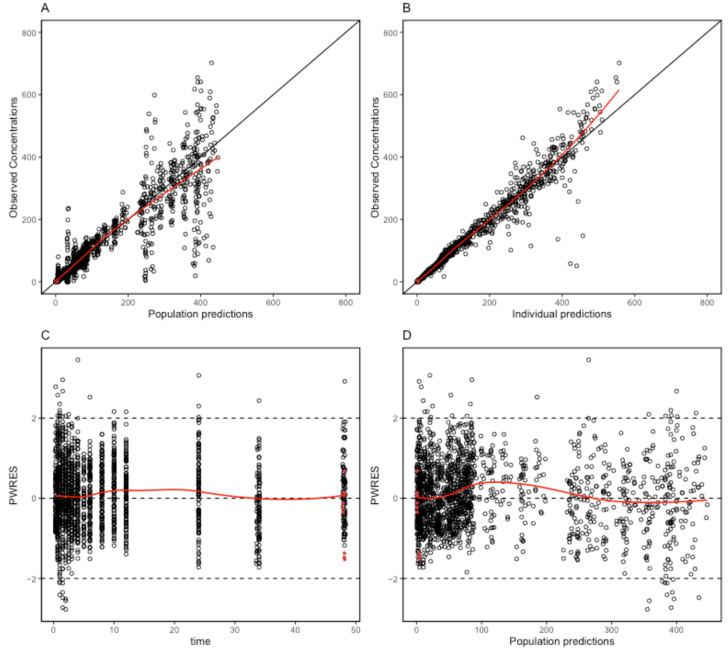
Goodness-of-fit plots of the final population pharmacokinetic model for empagliflozin. (**A**) Population predicted concentrations (PRED) against observed plasma concentration; (**B**) individual-predicted concentrations (IPRED) against observed plasma concentration; (**C**) time against conditional weighted residuals; (**D**) PRED against conditional weighted residuals. Red dots mean the data are not censored.

**Figure 3:**
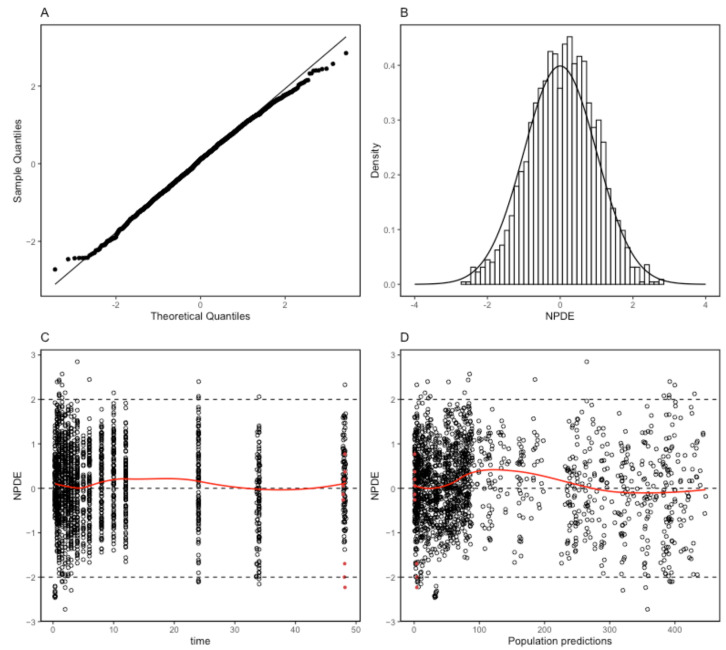
Normalized prediction distribution error (NPDE) metrics for the population pharmacokinetic model of empagliflozin. Normal Q–Q plot for NPDE (**A**), distribution of NPDE (**B**), and NPDE versus time after the first dose (**C**) and versus predicted concentrations (**D**). Red dots mean the data are not censored.

**Figure 4:**
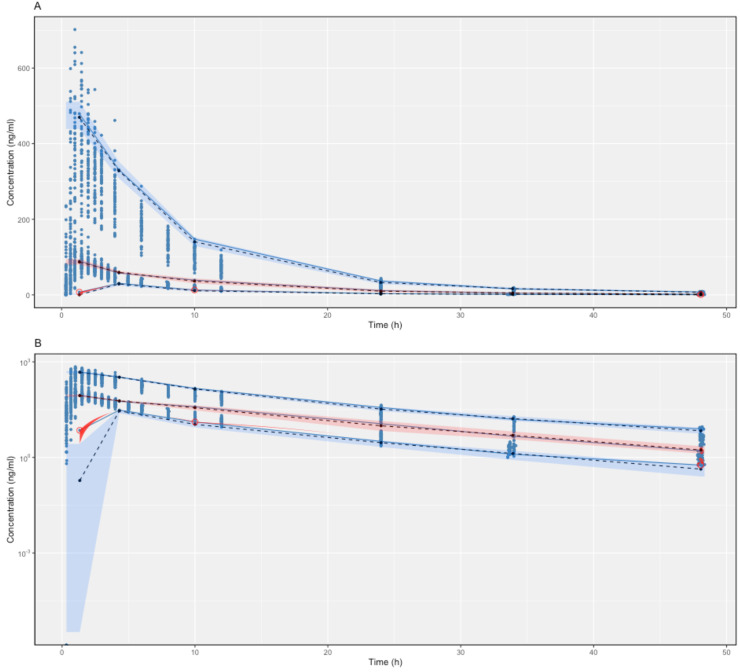
Linear scale (**A**) and semi-log scale (**B**) of visual predictive checks (VPCs) (500 simulations) of the final model for empagliflozin. The observed concentrations are depicted by dots. The solid blue lines indicate the 95th, 50th, and 5th percentiles of the observed concentrations. The black dashed lines indicate the predicted means. The blue shaded regions indicate the 95% confidence intervals for the predicted 5th and 95th percentiles. Pink shaded regions indicate the 95% confidence intervals for the predicted 50th percentiles. The red dots indicate the conserved data, and the red shaded regions indicate the outlined areas.

The proposed final model was robust and showed adequate performance based on GOF plots, VPC, and bootstrapping results. According to the GOF plots, there was no systematic bias to predictions and no trends were found in the population-weighted residuals (PWRES) versus time and population prediction (PRED) plots (Figure 2). The NPDE distribution and histogram agreed well with the standard normal distribution and density, which indicated that the model fit the individual data well (Figure 3). As shown in Figure 3C,D, there was no trend in NPDE versus time or versus PRED. According to the VPCs, most of the observed concentrations were within the 95% confidence intervals for the 5th, 50th, and 95th percentiles of the simulated concentration (Figure 4). The parameter estimates of the proposed final model were in close agreement with the median values from the bootstrap results (Table 2). The RSE values were small, and all the parameter values estimated with this final model were within 95% confidence intervals (CIs) of the bootstrap analysis results. Overall, the estimated IIV and IOV adequately described the observed variability in empagliflozin concentrations.

## 3. Discussion

Empagliflozin L-proline is a novel empagliflozin formulation that is produced using cocrystal technology to improve the chemical properties of drugs, such as stability, compressibility, flowability, and hygroscopicity [21,28,29,30,31]. Although the bioequivalence has been confirmed in previous studies by investigating the area under the concentration–time curve from 0 to last (AUC_last_) and the maximum concentration (C_max_) of two empagliflozin formulations after single-dose administration, the differences caused by the co-former may exist in the absorption phase, even if they are small. The population PK analysis is a mechanistic way of assessing PK properties and covariates. The purpose of this study was to compare the PK characteristics of two different empagliflozin formulations via population PK analysis and to identify potential covariates of PK parameters. 

According to the PK results in the clinical studies, the maximum values of time to maximum plasma concentration (T_max_) were the same; however, the minimum T_max_ values of empagliflozin L-proline were larger than that of empagliflozin for two dose strengths, which meant that empagliflozin L-proline took more time to reach T_max_, and also reflected that breaking down L-proline in the digestive system requires time [25,26]. However, the results of the population PK analysis showed that formulation differences did not affect PK parameters as covariates, which suggested that the difference in the absorption phase is not significantly meaningful and can be ignored. The PK parameters of both formulations were similar, indicating that the bioequivalence of the two formulations was once again confirmed by the population PK analysis. Only log-transformed body weight was found to be a covariate affecting both CL and V2.

In previous studies, rapid absorption after oral administration and biphasic elimination were confirmed, which indicated that the two-compartment model may be the best for describing the PK of empagliflozin [32]. Additionally, both the two-compartment model, with lagged first-order oral absorption and a lag time fixed at 0.5 h, and the three transit compartment model, with an estimated Mtt of 30 min, which indicates the average time spent by drug molecules traveling from the first transit compartment to the absorption compartment was approximately 30 min, could appropriately describe the plasma concentration data for empagliflozin [33,34,35,36]. In our study, the PK of empagliflozin was modeled as a two-compartment model with 4.79 transit compartments with first-order absorption and elimination and an Mtt of 37.8 min, which was similar to the value in the previous studies.

Notably, the empagliflozin concentration data from the second clinical study were acquired using fixed-dose combination therapy. Since metformin and empagliflozin have no common pathways for metabolism or any common transporters, PK interactions are less likely. The use of the second study data was thought to be appropriate. Moreover, the PK parameters were similar when modeling was performed with or without the second study’s data.

Individual parameters can vary among individuals and occasions; thus, both IIV and IOV need to be studied if data are collected on different occasions when developing a population PK model. IOV has long been recognized as being important for nonlinear mixed effects [37,38]. Since PK modeling was conducted in this study by using data from two crossover studies, the effects of period 1 and period 2 were reflected by the IOV.

This study had several limitations. First, in this study, we used data from two clinical studies to develop the model and performed an internal evaluation; however, because of the lack of independent datasets, an external evaluation was not included in this study. Data obtained from various patient populations are needed in the future to assess the external model. Second, the study data were obtained from only healthy subjects, which may limit the use of covariates. However, the effect of the formulation on population PK parameters was the primary focus of this study, and these results may not be affected by the subject’s health status. Third, our study subjects consisted of only a Korean population, and these results may not be generalizable to different racial groups. The PK characteristics of empagliflozin have been reported in Caucasian, Egyptian, Japanese, and Chinese subjects [13,14,15,16]. The extent of plasma exposure to empagliflozin in Korean subjects was similar to that in East Asian subjects (i.e., Chinese and Japanese), but it was greater than that in Egyptian and Caucasian subjects, which might be due to differences in the body weights of the ethnic groups. Future population PK studies in diverse racial groups should be conducted to elucidate the impact of race on the PK characteristics of empagliflozin.

## 4. Material and Methods

### 4.1. Data and Study Population

Data from two clinical studies (NCT03849495 and NCT03848637) were used in this study to develop the PK model and conduct population PK analysis. Both of the studies were randomized, open-label, two-period, two-sequence, crossover studies comparing the PK characteristics, safety and tolerability profiles of the two study drugs in healthy Korean subjects. Healthy subjects aged between 19 and 50 years with BMIs of 18–27 kg/m^2^ were recruited for both studies. Subjects without any clinically significant medical history, and without any clinically significant findings in physical examinations, vital signs, 12-lead electrocardiograms (ECG), and clinical laboratory test results were eligible for the studies. Subjects who could not abstain from alcohol, smoking, or drug use throughout the study were excluded. To ensure safety, subjects with any allergies to the study medications were also excluded. Adverse drug reactions were assessed throughout the study period, and the monitoring of physical examinations, vital signs, 12-lead ECG, and laboratory tests was also performed.

In the first study (NCT03849495), the study drugs used were 25 mg empagliflozin L-proline and the conventional 25 mg empagliflozin formulation [26]. In the second study (NCT03848637), the study drugs were fixed-dose combinations of 5 mg/1000 mg empagliflozin L-proline/metformin and 5 mg/1000 mg empagliflozin/metformin [25]. Demographic data, clinical laboratory data, and time–plasma concentration data were pooled to develop a population PK model.

Both studies were approved by the Ministry of Food and Drug Safety of the Republic of Korea and the Institutional Review Board of Seoul National University Hospital (Seoul, Republic of Korea). All the procedures were conducted in accordance with the International Conference on Harmonization Good Clinical Practice and the ethical principles of the Declaration of Helsinki. All the subjects provided written informed consent prior to enrollment.

### 4.2. Sample Collection and Analytical Methodology

Blood samples were collected before drug administration (0 h) and 0.33 h, 0.67 h, 1 h, 1.5 h, 2 h, 2.5 h, 3 h, 4 h, 6 h, 8 h, 10 h, 12 h, 24 h, 34 h, and 48 h after study’s drug administration in the first study. In the second study, blood samples were also collected at 3.5 h and 5 h. Thus, a total of 16 and 18 blood samples were collected from each healthy volunteer in the two clinical studies, respectively. Because the preclinical studies suggested that empagliflozin L-proline may be broken down into empagliflozin in the digestive system and absorbed as empagliflozin, the plasma concentrations of empagliflozin were tested in the two clinical studies and they were quantified by using developed and validated liquid chromatography with tandem mass spectrometry (LC–MS/MS) equipped with a Shimadzu UFLC system (Shimadzu, Kyoto, Japan) and an API5000(3) triple quadrupole mass spectrometer (SCIEX, CA, USA) in the first study. In the second study, the plasma concentrations of empagliflozin were quantified by using liquid chromatography–mass spectrometry (LC–MS) equipped with API 4000 (SCIEX; multiple reaction monitoring, electrospray ionization, and positive SCIEX mode). All experiments adhered to the standards of good laboratory practice as described previously [25,26]. The calibration ranges in these two studies were 2–1500 ng/mL and 0.5–150 ng/mL, respectively.

### 4.3. Base Model Development

The population PK analysis was conducted using a nonlinear mixed effect (NLME) modeling method with Monolix^®^ Suite 2021R1 (Lixoft, Antony, France) [39,40]. The stochastic approximation expectation maximization (SAEM) algorithm was implemented, and model qualification was performed using a likelihood ratio test, Bayesian information criterion (BIC), goodness-of-fit (GOF) diagnostic plots, and visual predictive checks (VPCs) [41]. Various compartment models with lag time, transit compartment, and Weibull absorption were established to construct the base PK model.

After the development of the base model, interindividual variability (IIV), interoccasion variability (IOV), and residual errors were added to the model [42]. Because of the crossover study design, the random variation in the population PK parameters was described by both the IIV and IOV, with all the individual parameters considered to be log-normally distributed. IIV and IOV were sequentially implemented, and the parameters were subsequently removed or fixed at low values when converging to zero. The exponential random effects terms for the IIV and IOV are shown in Formulas (2) and (3), respectively.

(2) $$P_{i}=\theta_{k}\times e^{\eta_{i}}$$

where P_i_ represents the parameter value k from individual i and θ_k_ describes the population value of parameter k. η_i_ denotes the difference between P_i_ and θ_k_.

(3) $$P_{i,\mathrm{occ}}=\theta_{k}\times e^{\eta_{i}+K_{\mathrm{kiq}}}$$

where P_i,occ_ is the individual parameter value k from individual i at occasion q that differs from the typical individual value by an additional random effect K_kiq_. An occasion was characterized as the time period from the start of an infusion until the start of the next administration. η_ki_ and K_kiq_ were assumed to be symmetrically distributed with a zero mean and a variance of σ^2^ and γ^2^, respectively.

Additionally, additive, proportional, and combined (additive and proportional) error models were utilized to explain the residual variability.

### 4.4. Covariate Analysis

From the base model (without covariate), the effects of the following 13 covariates on empagliflozin PK parameters were evaluated: treatment, age, height, weight, BMI, sex, glucose, protein, aspartate aminotransferase (AST), alkaline phosphatase (ALP), alanine transaminase (ALT), lactate dehydrogenase (LDH), and estimated glomerular filtration rate (eGFR) levels. The potential covariates were selected according to the plausible mechanism for their influence on PK variability and the previous population PK study of empagliflozin [33].

Data visualization was used to examine the relationship between intrinsic or extrinsic factors and subject-level PK parameters. The initial selection of covariates was guided by graphic inspection and biological plausibility. Potential covariates were tested further in Monolix. Conditional sampling for a stepwise approach based on correlation tests (COSSAC) was used for covariate searches [43]. The iterations of COSSAC alternated between forward selection and backward elimination, depending on the results of the correlation tests. The final model was derived from the covariate model by excluding covariates that had poor precision when the confidence interval had no effect.

### 4.5. Final Model Evaluation

The established empagliflozin population PK model was comprehensively evaluated via GOF plots, VPCs (n = 500), bootstrapping (n = 1000), and the normalized prediction distribution error (NPDE). GOF evaluation was performed by plotting the corresponding individual predictions (IPRED) and population predictions (PRED) against the observed values as well as the PRED and time against conditional weighted residual errors (CWRES).

Bootstrap resampling was used to assess the reliability and stability of the population PK model. A total of 1000 replicates were generated by repeated random sampling with replacement from the original dataset. Estimated parameter values and medians from the bootstrap procedure were compared with those estimated from the original dataset. All the processes of model evaluation and validation were performed using R version 4.2.0.

## 5. Conclusions

In this population PK study, a two-compartment combined transit compartment model with first-order absorption and elimination described the time–plasma concentration profile of empagliflozin well. The differences in formulation did not affect the absorption, distribution, or elimination characteristics of empagliflozin, and the subject’s body weight was the only significant covariate of the PK parameters. The study results can provide useful information for further empagliflozin studies and the clinical use of empagliflozin.

## Figures and Tables

**Table 1:** Demographic and biochemical information of the studied subjects (n = 54).

Physicochemical Parameters (Units)	Study A (n = 27)		Study B (n = 27)		All (n = 54)	
	Median [Min–Max]	Mean ± SD	Median [Min–Max]	Mean ± SD	Median [Min–Max]	Mean ± SD
Age (Year)	29 [20–50]	30 ± 7.38	27 [21–47]	30.22 ± 7.71	27.5 [20–50]	30.26 ± 7.48
Height (cm)	171.2 [156.3–186.3]	170.83 ± 7.01	172.4 [155.2–186.4]	171.60 ± 7.64	171.5 [155.2–186.4]	171.21 ± 7.28
Weight (kg)	72.3 [55.6–82.1]	69.62 ± 7.74	70.1 [56.1–83.4]	69.76 ± 8.05	72.1 [55.6–83.4]	69.69 ± 7.82
BMI (kg/m^2^)	24.2 [19.5–26.4]	23.84 ± 2.15	23.9 [19.8–26.9]	23.65 ± 1.95	24.1 [19.5–26.9]	23.75 ± 2.03
Glucose (mg/dL)	87 [79–96]	87.33 ± 5.67	87 [78–101]	88.07 ± 6.34	87 [78–101]	87.70 ± 5.97
Protein (g/dL)	6.7 [6.2–7.2]	6.67 ± 0.25	6.7 [6.3–7.4]	6.71 ± 0.26	6.7 [6.2–7.4]	6.69 ± 0.25
ALP (IU/L)	52 [30–83]	54.44 ± 14.19	59 [36–81]	58.63 ± 11.98	55.5 [30–83]	56.54 ± 13.18
AST (IU/L)	17 [12–27]	17.37 ± 4.1	17 [13–35]	17.85 ± 4.88	17 [12–35]	17.61 ± 4.47
ALT (IU/L)	17 [8–47]	18.56 ± 8.93	16 [7–64]	19.44 ± 12.20	16.5 [7–64]	19.00 ± 10.60
LDH (IU/L)	147 [128–219]	155.11 ± 23.04	147 [113–185]	145 ± 19.80	147 [113–219]	150.06 ± 21.88
eGFR (mL/min/1.73 m^2^)	115 [74.4–143.9]	111.57 ± 15.79	111.9 [74.2–138.7]	110.77 ± 15.74	112.35 [74.2–143.9]	111.17 ± 15.62

Min, minimum; Max, maximum; BMI, body mass index; ALP, alkaline phosphatase; AST, aspartate aminotransferase; ALT, alanine aminotransferase; LDH, lactate dehydrogenase; eGFR, estimated glomerular filtration rate using the Modification of Diet in Renal Disease (MDRD) equation.

**Table 2:** Estimates of population pharmacokinetics parameters.

Population Parameter (Unit)	Value	RSE (%)	Median of Bootstrap * (95% CI)	Shrinkage ^a^
Fixed effects				
Ktr (h^−1^)	9.19	12.4	8.94 (6.766–11.728)	3.79%
Mtt (h)	0.63	4.26	0.64 (0.526–0.725)	−0.239%
Ka (h^−1^)	0.26	3.42	0.25 (0.23–0.275)	1.7%
CL (L/h)	8.21	1.78	8.21 (7.945–8.465)	1.28%
V1 (L)	0.6	6.67	0.64 (0–1.889)	
V2 (L)	44.6	2.51	43.93 (41.255–47.171)	1.09%
Q (L/h)	4.92	5.09	4.66 (3.979–5.456)	
βCL_logWT	0.64	24.5	0.64 (0.366–0.926)	
βV2_logWT	0.57	30.3	0.54 (0.221–0.851)	
Interindividual variability (IIV) ^b^				
ωKa (%)	0.17 (17.12)	10.8	0.16 (0.116–0.208)	
ωCL (%)	0.12 (12.04)	10.9	0.12 (0.101–0.141)	
ωV2 (%)	0.13 (13.06)	12.0	0.13 (0.1–0.172)	
Interoccasion Variability (IOV) ^b^				
γKtr (%)	1.17 (171.20)	7.43	1.11 (0.858–1.334)	
γMtt (%)	0.42 (43.92)	7.15	0.44 (0.359–0.528)	
γKa (%)	0.029 (2.90)	34.3	0.04 (0.023–0.062)	
γCL (%)	0.043 (4.30)	16.7	0.04 (0.025–0.06)	
Residual variability				
b	0.16	2.01	0.16 (0.141–0.175)	

* from 1000 bootstrap resampling. ^a^ Shrinkage was calculated based on the variance in the Monolix. ^b^ The IIV and IOV are presented as SD (CV). RSE, relative standard error; CI, confidence interval; Ktr, identical transfer rate constant of the transit compartment model; Mtt, mean transit time for absorption; SD, standard deviation; CV, coefficient of variation.

## Data Availability

The individual deidentified participant data that support the published results in this study are available from the corresponding author or sponsor upon reasonable request.

## References

[1] Ha K.H., Lee K.A., Han K.D., Moon M.K., Kim D.J. (2023). Diabetes screening in South Korea: A new estimate of the number needed to screen to detect diabetes. Korean J. Intern. Med..

[2] Marin-Penalver J.J., Martin-Timon I., Sevillano-Collantes C., Del Canizo-Gomez F.J. (2016). Update on the treatment of type 2 diabetes mellitus. World J. Diabetes.

[3] Lee M.K., Lee S.Y., Sohn S.Y., Ahn J., Han K., Lee J.H. (2023). Type 2 Diabetes and Its Association With Psychiatric Disorders in Young Adults in South Korea. JAMA Netw. Open.

[4] Tran L., Zielinski A., Roach A.H., Jende J.A., Householder A.M., Cole E.E., Atway S.A., Amornyard M., Accursi M.L., Shieh S.W. (2015). Pharmacologic treatment of type 2 diabetes: Oral medications. Ann. Pharmacother..

[5] Mao T., Meng Q., Zhang H., Zhang J.J., Shi S., Guan Z., Jiang X., Zhang F., Lei H., Lin X. (2023). 760-P: Discovery of GSBR-1290, a Highly Potent, Orally Available, Novel Small Molecule GLP-1 Receptor Agonist. Diabetes.

[6] Rieg T., Vallon V. (2018). Development of SGLT1 and SGLT2 inhibitors. Diabetologia.

[7] Kavanagh O.N., Croker D.M., Walker G.M., Zaworotko M.J. (2019). Pharmaceutical cocrystals: From serendipity to design to application. Drug Discov. Today.

[8] Tamura H., Kondo Y., Ito K., Hasebe M., Satoh S., Terauchi Y. (2022). Comparison of the effects of empagliflozin and glimepiride on endothelial function in patients with type 2 diabetes: A randomized controlled study. PLoS ONE.

[9] Hierro-Bujalance C., Garcia-Alloza M. (2024). Empagliflozin reduces brain pathology in Alzheimer’s disease and type 2 diabetes. Neural Regen. Res..

[10] Chen S., Overberg K., Ghouse Z., Hollmann M.W., Weber N.C., Coronel R., Zuurbier C.J. (2024). Empagliflozin mitigates cardiac hypertrophy through cardiac RSK/NHE-1 inhibition. Biomed. Pharmacother..

[11] Lv C., Hu C., Zhu C., Wan X., Chen C., Ji X., Qin Y., Lu L., Guo X. (2024). Empagliflozin alleviates the development of autoimmune myocarditis via inhibiting NF-kappaB-dependent cardiomyocyte pyroptosis. Biomed. Pharmacother..

[12] Sheng W., Yu J., Zhang H., Zhang J. (2024). Empagliflozin attenuates inflammation levels in autoimmune myocarditis through the STAT3 pathway and macrophage phenotype transformation. Mol. Immunol..

[13] Li X., Liu L., Deng Y., Li Y., Zhang P., Wang Y., Xu B. (2020). Pharmacokinetics and bioequivalence of a generic empagliflozin tablet versus a brand-named product and the food effects in healthy Chinese subjects. Drug Dev. Ind. Pharm..

[14] Ayoub B.M., Mowaka S., Elzanfaly E.S., Ashoush N., Elmazar M.M., Mousa S.A. (2017). Pharmacokinetic Evaluation of Empagliflozin in Healthy Egyptian Volunteers Using LC-MS/MS and Comparison with Other Ethnic Populations. Sci. Rep..

[15] Sarashina A., Koiwai K., Seman L.J., Yamamura N., Taniguchi A., Negishi T., Sesoko S., Woerle H.J., Dugi K.A. (2013). Safety, tolerability, pharmacokinetics and pharmacodynamics of single doses of empagliflozin, a sodium glucose cotransporter 2 (SGLT2) inhibitor, in healthy Japanese subjects. Drug Metab. Pharmacokinet..

[16] Seman L., Macha S., Nehmiz G., Simons G., Ren B., Pinnetti S., Woerle H.J., Dugi K. (2013). Empagliflozin (BI 10773), a Potent and Selective SGLT2 Inhibitor, Induces Dose-Dependent Glucosuria in Healthy Subjects. Clin. Pharmacol. Drug Dev..

[17] Yadav A.V., Shete A.S., Dabke A.P., Kulkarni P.V., Sakhare S.S. (2009). Co-crystals: A novel approach to modify physicochemical properties of active pharmaceutical ingredients. Indian J. Pharm. Sci..

[18] Gadade D.D., Pekamwar S.S. (2016). Pharmaceutical Cocrystals: Regulatory and Strategic Aspects, Design and Development. Adv. Pharm. Bull..

[19] Shan N., Perry M.L., Weyna D.R., Zaworotko M.J. (2014). Impact of pharmaceutical cocrystals: The effects on drug pharmacokinetics. Expert Opin. Drug Metab. Toxicol..

[20] Guo M., Sun X., Chen J., Cai T. (2021). Pharmaceutical cocrystals: A review of preparations, physicochemical properties and applications. Acta Pharm. Sin. B.

[21] Emami S., Siahi-Shadbad M., Adibkia K., Barzegar-Jalali M. (2018). Recent advances in improving oral drug bioavailability by cocrystals. Bioimpacts.

[22] Cui W., He Z., Zhang Y., Fan Q., Feng N. (2019). Naringenin Cocrystals Prepared by Solution Crystallization Method for Improving Bioavailability and Anti-hyperlipidemia Effects. AAPS PharmSciTech.

[23] Ma S., Liu Z., Pan J., Zhang S., Zhou W. (2017). A concise and practical stereoselective synthesis of ipragliflozin L-proline. Beilstein J. Org. Chem..

[24] Cho H.J., Woo M.R., Cho J.H., Kim Y.I., Choi H.G. (2022). Novel dapagliflozin di-L-proline cocrystal-loaded tablet: Preparation, physicochemical characterization, and pharmacokinetics in beagle dogs and mini-pigs. Pharm. Dev. Technol..

[25] Lee H., Chung J.Y., Yu K.S., Park S.J., Lee S. (2023). Pharmacokinetic Comparison Between a Fixed-Dose Combination of Empagliflozin L-Proline/Metformin and Empagliflozin/Metformin in Healthy Korean Subjects. Clin. Pharmacol. Drug Dev..

[26] Jiang X., Bae S., Yoon D.Y., Park S.J., Oh J., Cho J.Y., Yu K.S. (2023). Comparison of the Pharmacokinetics, Safety, and Tolerability of Two Empagliflozin Formulations in Healthy Korean Subjects. Drug Des. Devel. Ther..

[27] Zandvliet A.S., Schellens J.H., Beijnen J.H., Huitema A.D. (2008). Population pharmacokinetics and pharmacodynamics for treatment optimization in clinical oncology. Clin. Pharmacokinet..

[28] Aguillon A.R., Mascarello A., Segretti N.D., de Azevedo H.F.Z., Guimaraes C.R.W., Miranda L.S.M., de Souza R.O.M.A. (2018). Synthetic Strategies toward SGLT2 Inhibitors. Org. Process Res. Dev..

[29] Bolla G., Nangia A. (2016). Pharmaceutical cocrystals: Walking the talk. Chem. Commun..

[30] Shinozaki T., Ono M., Higashi K., Moribe K. (2019). A Novel Drug-Drug Cocrystal of Levofloxacin and Metacetamol: Reduced Hygroscopicity and Improved Photostability of Levofloxacin. J. Pharm. Sci..

[31] Kale D.P., Ugale B., Nagaraja C.M., Dubey G., Bharatam P.V., Bansal A.K. (2019). Molecular Basis of Water Sorption Behavior of Rivaroxaban-Malonic Acid Cocrystal. Mol. Pharm..

[32] Scheen A.J. (2014). Pharmacokinetic and pharmacodynamic profile of empagliflozin, a sodium glucose co-transporter 2 inhibitor. Clin. Pharmacokinet..

[33] Riggs M.M., Staab A., Seman L., MacGregor T.R., Bergsma T.T., Gastonguay M.R., Macha S. (2013). Population pharmacokinetics of empagliflozin, a sodium glucose cotransporter 2 inhibitor, in patients with type 2 diabetes. J. Clin. Pharmacol..

[34] Baron K.T., Macha S., Broedl U.C., Nock V., Retlich S., Riggs M. (2016). Population Pharmacokinetics and Exposure-Response (Efficacy and Safety/Tolerability) of Empagliflozin in Patients with Type 2 Diabetes. Diabetes Ther..

[35] Mondick J., Riggs M., Kaspers S., Soleymanlou N., Marquard J., Nock V. (2018). Population Pharmacokinetic-Pharmacodynamic Analysis to Characterize the Effect of Empagliflozin on Renal Glucose Threshold in Patients With Type 1 Diabetes Mellitus. J. Clin. Pharmacol..

[36] Riggs M.M., Seman L.J., Staab A., MacGregor T.R., Gillespie W., Gastonguay M.R., Woerle H.J., Macha S. (2014). Exposure-response modelling for empagliflozin, a sodium glucose cotransporter 2 (SGLT2) inhibitor, in patients with type 2 diabetes. Br. J. Clin. Pharmacol..

[37] Kristoffersson A.N., Friberg L.E., Nyberg J. (2015). Inter occasion variability in individual optimal design. J. Pharmacokinet. Pharmacodyn..

[38] U.S. Department of Health and Human Services Food and Drug Administration (2022). Population Pharmacokinetics Guidance for Industry.

[39] Lindstrom M.L., Bates D.M. (1990). Nonlinear mixed effects models for repeated measures data. Biometrics.

[40] Sheiner L.B., Ludden T.M. (1992). Population pharmacokinetics/dynamics. Annu. Rev. Pharmacol. Toxicol..

[41] Savic R.M., Mentre F., Lavielle M. (2011). Implementation and evaluation of the SAEM algorithm for longitudinal ordered categorical data with an illustration in pharmacokinetics-pharmacodynamics. AAPS J..

[42] Abrantes J.A., Jonsson S., Karlsson M.O., Nielsen E.I. (2019). Handling interoccasion variability in model-based dose individualization using therapeutic drug monitoring data. Br. J. Clin. Pharmacol..

[43] Ayral G., Si Abdallah J.F., Magnard C., Chauvin J. (2021). A novel method based on unbiased correlations tests for covariate selection in nonlinear mixed effects models: The COSSAC approach. CPT Pharmacomet. Syst. Pharmacol..

